# O Paradoxo da Obesidade na Insuficiência Cardíaca Depende da Aptidão Cardiorrespiratória?

**DOI:** 10.36660/abc.20200522

**Published:** 2020-10-13

**Affiliations:** 1 University of Eastern Finland Institute of Clinical Medicine Kuopio Finlândia Institute of Clinical Medicine, University of Eastern Finland, Kuopio - Finlândia; 2 Central Finland Health Care District Departamento de Medicina Jyväskylä Finlândia Departamento de Medicina, Central Finland Health Care District, Jyväskylä - Finlândia; 3 University of Eastern Finland Institute of Public Health and Clinical Nutrition Kuopio Finlândia Institute of Public Health and Clinical Nutrition, University of Eastern Finland, Kuopio - Finlândia; 4 University of Bristol University Hospitals Bristol NHS Foundation Trust National Institute for Health Research Bristol Biomedical Research Centre Bristol Reino Unido National Institute for Health Research Bristol Biomedical Research Centre, University Hospitals Bristol NHS Foundation Trust and University of Bristol, Bristol - Reino Unido; 5 University of Bristol Bristol Medical School Translational Health Sciences Bristol Reino Unido Musculoskeletal Research Unit, Translational Health Sciences, Bristol Medical School, University of Bristol, Learning & Research Building (Level 1), Southmead Hospital, Bristol - Reino Unido

**Keywords:** Insuficiência Cardíaca, Obesidade, Aptidão Cardiorrespiratória, Índice de Massa Corporal, Exercício, Hospitalização, Morbidade e Mortalidade

A insuficiência cardíaca (IC) é uma ameaça global à saúde pública, associada ao aumento de hospitalizações, morbidade, mortalidade e altos custos econômicos.^1^ Entretanto, a IC, a qual tem a doença cardíaca isquêmica como doença subjacente comum, é uma condição pelo menos parcialmente evitável. A aptidão cardiorrespiratória (ACR) é um índice dos níveis habituais de atividade física e é considerado o padrão ouro para a capacidade de exercício aeróbico. A aptidão cardiorrespiratória, um componente importante da aptidão física e medida diretamente pelo consumo de oxigênio de pico (VO_2_ pico) e a inclinação da eficiência ventilatória (inclinação VE/VCO_2_), foi identificada como um dos preditores mais importantes dos desfechos de saúde e sobrevivência.^2^ Relatamos anteriormente que o VO_2_ pico, quando diretamente avaliado, está forte, independente e inversamente relacionado a um risco reduzido de condições cardiometabólicas, como IC,^2^^,^^3^ fibrilação atrial,^3^^,^^4^ fibrilação ventricular^5^ diabetes,^6^ bem como mortalidade por doenças cardiovasculares (DCV).^7^^,^^8^ Nosso recente estudo de base populacional baseado no *UK Biobank* sugere que a ACR é um forte indicador de risco para mortalidade e a adição da ACR ao escore dos fatores de risco convencional melhorou a discriminação geral do risco de mortalidade e, mais importante, o valor preditivo da ACR variou através dos níveis de alguns fatores de risco relevantes, incluindo idade, sexo e tabagismo.^8^ Este fato é um indicador de que a ACR é uma ferramenta potencialmente valiosa de avaliação de risco na clínica prática.

Sabe-se que a obesidade, medida pelo índice de massa corporal (IMC), está relacionada ao desenvolvimento de desfechos cardiovasculares. No entanto, o efeito combinado da obesidade e da ACR no risco de IC futura ainda requer estudos adicionais. É de relevância clínica entender se a ACR atenua a associação da obesidade com o risco posterior de IC devido a outras DCV subjacentes.^9^ A maioria dos estudos anteriores sobre IMC mais alto, outros parâmetros de obesidade e risco de IC não levou em consideração as diferenças nos níveis de ACR. Embora o IMC alto seja um fator de risco para IC, há achados de uma relação não-linear entre os resultados de IMC e DCV em pacientes com IC, o que indica uma relação incomum. Em pacientes com IC estabelecida, evidências acumuladas sugerem que indivíduos com sobrepeso e obesidade (IMC mais alto) apresentaram melhor sobrevida em comparação com aqueles com IMC normal, um conceito conhecido que foi chamado de “paradoxo da obesidade” ou “epidemiologia reversa”.^10^ Alguns mecanismos têm sido propostos para explicar o paradoxo da obesidade da IC, que inclui a terapia da IC ser mais eficaz em pacientes obesos. Foi relatado que a ACR também pode desempenhar um papel, mitigando ou negando o “paradoxo da obesidade”.^10^^,^^11^

Há evidências limitadas sobre a associação e interações entre ACR, IMC, IC e desfechos da IC. Essa foi a justificativa para o novo estudo de Moreira et al. publicado na edição recente da revista, que teve como objetivo investigar o impacto da tolerância ao exercício e da capacidade cardiorrespiratória no paradoxo da obesidade em pacientes com IC com baixa fração de ejeção.^12^ Todos os pacientes encaminhados para os Ambulatórios de Insuficiência Cardíaca foram submetidos a avaliação clínica com coleta laboratorial, eletro- e ecocardiografia, e teste de esforço cardiopulmonar. Um teste de exercício cardiopulmonar máximo em esteira limitado por sintomas foi realizado utilizando o protocolo de Bruce modificado (esteira GE Marquette Series 2000). A ventilação minuto, consumo de oxigênio e a produção de dióxido de carbono foram avaliadas utilizando-se um analisador de gases respiração por respiração. Foram incluídos 282 pacientes com IC (75,5% do sexo masculino), com média de idade de 53,7 anos e IMC de 28,6 kg/m^2^. Os pacientes foram acompanhados por 60 meses para um *endpoint* composto combinado, consistindo em morte cardíaca, transplante cardíaco urgente ou necessidade de suporte circulatório mecânico.^12^ Curiosamente, pacientes com IMC maior apresentaram FEVE mais alta, mas menor inclinação da eficiência ventilatória (VE / VCO_2_).^12^ A análise de associação mostrou que o IMC, a inclinação VE / VCO_2_ e o VO_2_ pico são indicadores do desfecho primário. Entretanto, a associação entre IMC e desfecho foi atenuada para nula quando os pacientes foram agrupados em ACR baixa e alta (com base na VE / VCO_2_) e após o controle da VE / VCO_2_ ou VO_2_ pico.

Consistente com os achados deste estudo, Chase et al. demonstraram anteriormente que a inclinação VE / VCO_2_ mantinha seu valor prognóstico independentemente do IMC em pacientes com IC.^13^ No entanto, quando a inclinação VE / VCO_2_ ou o VO_2_ pico foram levados em consideração em suas análises, a associação entre o IMC e os desfechos cardíacos não foi significativa. Além disso, quando os pacientes foram agrupados e analisados de acordo com os subgrupos da ACR, o IMC não foi relacionado aos desfechos.^13^

Dado que o risco absoluto de resultados adversos de DCV depende muito da idade, o emprego de uma única métrica relativa não pode permitir a quantificação de diferenças absolutas de risco em idades crescentes com diferentes níveis de IMC. Zaccardi et al.,^14^ Recentemente, sugeriu uma nova abordagem para avaliar a interação para estimar a expectativa de vida residual entre os níveis de ACR e IMC.^14^ Com base na grande coorte do Reino Unido do *Biobank*, incluindo 474.919 participantes, os autores demonstraram que os participantes com um ritmo acelerado de caminhada têm uma expectativa de vida mais longa no espectro do IMC e outras medidas de obesidade, fornecendo dados adicionais de evidências de que o ritmo de caminhada, como marcador do nível de condicionamento físico, é um indicador facilmente disponível do estado de saúde.

Os pontos fortes do presente estudo incluem o uso de uma amostra clinicamente relevante com base em pacientes com IC de alto risco e a avaliação de medidas de ACR utilizando análise confiável de gases respiratórios, que é um método de avaliação mais objetivo e quantitativo da ACR. Várias limitações incluem (i) o pequeno tamanho da amostra; (ii) incapacidade de ajuste para muitos fatores de confusão em potencial; (iii) falta de dados sobre medicamentos, biomarcadores e padrões de atividade física durante o seguimento, o que poderia ter pelo menos parcialmente influenciado os níveis de ACR e IMC; (iv) falta de análises formais de previsão de risco, pois as medidas de associação são insuficientes para fazer julgamentos clínicos sobre a relevância prognóstica de uma exposição; e (v) o potencial de diluição de regressão, dada a ausência de medidas repetidas de ACR, devido ao potencial de alterações nos níveis de ACR devido a erros nas medições e alterações no curso clínico da doença. Em nossos sub-estudos de reprodutibilidade das medidas de ACR no estudo de coorte prospectivo *Kuopio Ischemic Heart Disease* (KIHD), demonstramos uma alta variabilidade intrapessoal nos níveis de ACR medidos com muitos anos de diferença (razão de diluição de regressão = 0,58),^15^ o que sugere que as análises utilizando apenas medições basais únicas da ACR poderiam subestimar as associações com os desfechos. Em geral, essas novas descobertas destacam o fato de que existe um “paradoxo da obesidade” em pacientes com IC e há uma interação entre obesidade, ACR e desfechos na IC. A aptidão cardiorrespiratória medida pela VE/VCO_2_ ou VO_2_ pico pareceu negar o paradoxo da obesidade.^12^

A ACR é influenciada por fatores genéticos e ambientais; aproximadamente 50% da variação da ACR foi atribuída a fatores hereditários, com a contribuição de fatores herdados para a resposta da ACR à atividade física e ao treinamento físico.^7^^,^^8^^,^^11^ Também depende de vários fatores, como status de saúde e condicionamento físico basais, tipo, duração e intensidade da AF. O nível de ACR também é um indicador de uma cadeia de múltiplos processos fisiológicos que incluem: ventilação pulmonar e função vascular, função ventricular direita e esquerda, capacidade da vasculatura em transportar sangue do coração com eficiência para outros órgãos, atendendo suas necessidades de oxigênio, a capacidade das células musculares em utilizar o oxigênio e os nutrientes essenciais e a capacidade de ativar todas as fibras musculares necessárias para o movimento corporal.^11^ O volume sistólico ventricular esquerdo, a frequência cardíaca máxima e a diferença arteriovenosa de oxigênio no exercício determinaram essencialmente os níveis de ACR. A função ventricular esquerda é uma medida-chave da IC e o nível da ACR pode refletir a função do VE. Como a ACR está relacionada à integração da função do corpo humano sob condições de estresse fisiológico, ela pode ser empregada como um indicador muito preciso do risco de IC, refletindo o estado funcional do corpo inteiro em pacientes com IC existente. Dado o papel central que a função cardíaca normal desempenha na definição da capacidade aeróbica máxima, doença ou disfunção que afeta negativamente o débito cardíaco, também comprometerá o VO_2_ máximo. O treinamento aeróbico de alta intensidade é seguro e eficaz para melhorar a capacidade funcional em muitas populações de pacientes com problemas cardíacos.^11^ A participação em um programa de treinamento aeróbico de longo prazo produz uma série de adaptações cardiovasculares morfológicas e fisiológicas positivas em indivíduos aparentemente saudáveis, independentemente de idade e sexo. Adaptações morfológicas comumente relatadas associadas ao treinamento aeróbico regular é a dilatação do ventrículo esquerdo (aumento do diâmetro diastólico final) e hipertrofia (aumento da espessura da parede), conhecido como remodelamento cardíaco induzido pelo treinamento com exercícios.^16^

Os achados atuais lançam mais luz sobre uma possível interação entre ACR, obesidade e desfechos na IC. No entanto, dadas as limitações do pequeno tamanho da amostra e a incapacidade de se ajustar às covariáveis relevantes, esses achados observacionais do estudo precisam de cautela ao serem interpretados. Os achados aumentam o arsenal de evidências acumuladas de que a ACR (usando o VO_2_ pico e medidas de eficiência ventilatória) é uma ferramenta clinicamente útil na avaliação de risco de IC. Altos níveis de ACR podem ser alcançados por meio de atividade física regular e isso deve ser promovido extensivamente através de abordagens em toda a população. Os benefícios de saúde associados à atividade física regular, que incluem componentes aeróbicos e de treinamento de força, não podem ser enfatizados em excesso.^11^^,^^14^^,^^16^ Os esforços para melhorar a ACR com um peso corporal saudável podem se tornar uma parte padrão dos encontros clínicos para o tratamento da IC com baixa fração de ejeção.

## References

[1] 1. Yancy CW, Jessup M, Bozkurt B, Butler J, Casey DE, Jr., Drazner MH, et al. 2013 ACCF/AHA guideline for the management of heart failure: a report of the American College of Cardiology Foundation/American Heart Association Task Force on Practice Guidelines. J Am Coll Cardiol. 2013;62(16):e147-239. 10.1016/j.jacc.2013.05.019 23747642

[2] 2. Khan H, Kunutsor S, Rauramaa R, Savonen K, Kalogeropoulos AP, Georgiopoulou VV, et al. Cardiorespiratory fitness and risk of heart failure: a population-based follow-up study. Eur J Heart Fail. 2014;16(2):180-8. 10.1111/ejhf.37 24464981

[3] 3. Khan H, Kunutsor SK, Rauramaa R, Merchant FM, Laukkanen JA. Long-Term Change in Cardiorespiratory Fitness in Relation to Atrial Fibrillation and Heart Failure (from the Kuopio Ischemic Heart Disease Risk Factor Study). Am J Cardiol. 2018;121(8):956-60. 10.1016/j.amjcard.2018.01.003 29472009

[4] 4. Khan H, Kella D, Rauramaa R, Savonen K, Lloyd MS, Laukkanen JA. Cardiorespiratory fitness and atrial fibrillation: A population-based follow-up study. Heart Rhythm. 2015;12(7):1424-30. 10.1016/j.hrthm.2015.03.024 25778429

[5] 5. Laukkanen JA, Lavie CJ, Khan H, Kurl S, Kunutsor SK. Cardiorespiratory Fitness and the Risk of Serious Ventricular Arrhythmias: A Prospective Cohort Study. Mayo Clin Proc. 2019;94(5):833-41. 10.1016/j.mayocp.2018.11.027 30935710

[6] 6. Zaccardi F, O’Donovan G, Webb DR, Yates T, Kurl S, Khunti K, et al. Cardiorespiratory fitness and risk of type 2 diabetes mellitus: A 23-year cohort study and a meta-analysis of prospective studies. Atherosclerosis. 2015;243(1):131-7. 10.1016/j.atherosclerosis.2015.09.016 26386209

[7] 7. Laukkanen JA, Zaccardi F, Khan H, Kurl S, Jae SY, Rauramaa R. Long-term Change in Cardiorespiratory Fitness and All-Cause Mortality: A Population-Based Follow-up Study. Mayo Clin Proc. 2016;91(9):1183-8. 10.1016/j.mayocp.2016.05.014 27444976

[8] 8. Laukkanen JA, Kunutsor SK, Yates T, Willeit P, Kujala UM, Khan H, et al. Prognostic Relevance of Cardiorespiratory Fitness as Assessed by Submaximal Exercise Testing for All-Cause Mortality: A UK Biobank Prospective Study. Mayo Clinic Proceedings. 2020;95(5):867-78. 10.1016/j.mayocp.2019.12.030 32370851

[9] 9. Laukkanen JA, Kunutsor SK. Fitness Equals Longer Life Expectancy Regardless of Adiposity Levels. Mayo Clin Proc. 2019;94(6):942-5. 10.1016/j.mayocp.2019.04.016 31171130

[10] 10. Lavie CJ, Sharma A, Alpert MA, De Schutter A, Lopez-Jimenez F, Milani RV, et al. Update on Obesity and Obesity Paradox in Heart Failure. Prog Cardiovasc Dis. 2016;58(4):393-400. 10.1016/j.pcad.2015.12.003 26721180

[11] 11. Laukkanen JA, Kunutsor SK, Ozemek C, Makikallio T, Lee DC, Wisloff U, et al. Cross-country skiing and running's association with cardiovascular events and all-cause mortality: A review of the evidence. Prog Cardiovasc Dis. 2019;62(6):505-14. 10.1016/j.pcad.2019.09.001 31505192

[12] 12. Moreira RI, Silva TP, Gonçalves AV, Feliciano J, Rio P, Soares R, et al. Impact of Cardiorespiratory Fitness on the Obesity Paradox in Heart Failure with Reduced Ejection Fraction. Arq Bras Cardiol. 2020; 115(4):639-645. 10.36660/abc.20190337 PMC8386967 33111862

[13] 13. Chase P, Arena R, Myers J, Abella J, Peberdy MA, Guazzi M, et al. Relation of the prognostic value of ventilatory efficiency to body mass index in patients with heart failure. Am J Cardiol. 2008;101(3):348-52. 10.1016/j.amjcard.2007.08.042 18237598

[14] 14. Zaccardi F, Davies M, Khunti K, Yates T. Comparative relevance of physical fitness and adiposity on life expectancy: A UK Biobank observational study. Mayo Clinic proceedings. 2020;95(5):867-78. 10.1016/j.mayocp.2018.10.029 31079962

[15] 15. Kunutsor SK, Makikallio TH, Araujo CGS, Jae SY, Kurl S, Laukkanen JA. Cardiorespiratory fitness is not associated with risk of venous thromboembolism: a cohort study. Scand Cardiovasc J. 2019;53(5):255-8. 10.1080/14017431.2019.1630748 31180252

[16] 16. Lavie CJ, Arena R, Swift DL, Johannsen NM, Sui X, Lee DC, et al. Exercise and the cardiovascular system: clinical science and cardiovascular outcomes. Circ Res. 2015;117(2):207-19. 10.1161/CIRCRESAHA.117.305205 PMC4493772 26139859

