# Supplementary material for: O Paradoxo da Obesidade na Insuficiência Cardíaca Depende da Aptidão Cardiorrespiratória?
Source: Arq Bras Cardiol. 2020 Oct 13;115(4):646–8. [Article in Portuguese] doi: 10.36660/abc.20200522 (PMC8386983; doi:10.36660/abc.20200522)
Supplement: Translation

# Translation: Is the Obesity Paradox in Heart Failure Dependent on Cardiorespiratory Fitness?

**Affiliations:** 1 Kuopio Finland Institute of Clinical Medicine, University of Eastern Finland, Kuopio - Finland; 2 Jyväskylä Finland Department of Medicine, Central Finland Health Care District, Jyväskylä - Finland; 3 Kuopio Finland Institute of Public Health and Clinical Nutrition, University of Eastern Finland, Kuopio - Finland; 4 Bristol UK National Institute for Health Research Bristol Biomedical Research Centre, University Hospitals Bristol NHS Foundation Trust and University of Bristol, Bristol - UK; 5 Bristol UK Musculoskeletal Research Unit, Translational Health Sciences, Bristol Medical School, University of Bristol, Learning & Research Building (Level 1), Southmead Hospital, Bristol - UK

**Keywords:** Heart Failure, Obesity, Cardiorespiratory Fitness, Body Mass Index, Exercise, Hospitalization, Morbity and Mortality

Heart failure (HF) is a global public health menace which is associated with increased hospitalization, morbidity, mortality and high economic costs.^1^ However, HF which has ischemic heart disease as a common underlying disease, is a preventable condition. Cardiorespiratory fitness (CRF) is an index of habitual physical activity levels and is considered the gold standard for aerobic exercise capacity. Cardiorespiratory fitness, an important component of physical fitness and directly measured by peak oxygen uptake (pVO_2_), and ventilatory efficiency slope (VE/VCO_2_ slope), has been identified as one of the most important predictors of health outcomes and survival.^2^ We have previously reported that directly assessed pVO_2_ is strongly, independently and inversely related to a lowered risk of cardiometabolic conditions such as HF,^2^^,^^3^ atrial fibrillation,^3^^,^^4^ ventricular fibrillation,^5^ diabetes^6^ as well as mortality from cardiovascular diseases (CVDs).^7^^,^^8^ Our recent population-based study based on UK Biobank suggests that CRF is a strong risk indicator for mortality and addition of CRF to a conventional risk factor score improved the overall discrimination of mortality risk and, more importantly, the predictive value of CRF varied across levels of some relevant risk factors, including age, sex, and smoking.^8^ This is an indicator of the potential of CRF to be a valuable risk assessment tool in clinical practice.

It is well-known that obesity, as measured by body mass index (BMI), is related to the development of cardiovascular outcomes. However, the combined effect of obesity and CRF on the risk of future HF still requires further study. It is of clinical relevance to understand if CRF attenuates the association of obesity with later risk of HF due to other underlying CVDs.^9^ The majority of previous studies on higher BMI, other obesity parameters, and HF risk have not taken into account the differences in CRF levels. Though high BMI is a risk factor for HF, there are findings of a non-linear relationship between BMI and CVD outcomes in HF patients which indicates an unusual relationship. In patients with established HF, accumulating evidence suggests that overweight and obese (higher BMI) individuals have improved survival compared to those with normal BMI, a known concept which has been called the “obesity paradox” or “reverse epidemiology”.^10^ Some mechanisms have been proposed to explain the HF obesity paradox which includes HF therapy being more effective in obese patients. It has been reported that CRF might play also play a role by either mitigating or negating the “obesity paradox”.^10^^,^^11^

There is limited evidence on the association and interactions between CRF, BMI, HF and HF outcomes. This was the rationale for the new study by Moreira et al. published in the recent issue of the journal, which aimed to investigate the impact of exercise tolerance and cardiorespiratory capacity on the obesity paradox among HF patients with low ejection fraction.^12^ All patients referred to the Heart Failure Clinics underwent clinical evaluation with collection of laboratory, electro- and echocardiographic, and cardiopulmonary exercise test data. A maximal symptom-limited treadmill cardiopulmonary exercise test was performed using the modified Bruce protocol (GE Marquette Series 2000 treadmill). Minute ventilation, oxygen uptake, and carbon dioxide production were acquired using a breath-by-breath gas analyzer. A total of 282 HF patients were included (75.5% male) with a mean age of 53.7 years and BMI of 28.6 kg/m^2^. Patients were followed-up for 60 months for a combined composite endpoint comprising of cardiac death, urgent heart transplant, or need for mechanical circulatory support.^12^ Interestingly, patients with higher BMI had higher LVEF but lower ventilatory efficiency (VE/VCO_2_) slope.^12^ Association analysis showed BMI, VE/VCO_2_ slope and pVO_2_ to be each an indicator of the primary outcome. However, the association between BMI and outcome was attenuated to null when patients were grouped into low and high CRF (based on VE/VCO_2_) and after controlling for VE/VCO_2_ or pVO_2_.

Consistent with findings of this study, Chase et al. have earlier demonstrated that VE/VCO_2_ slope maintained its prognostic value irrespective of BMI in patients with HF.^13^ However, when either VE/VCO_2_ slope or pVO_2_ were taken into account in their analyses, the association between BMI and cardiac outcomes were not significant. Moreover, when the patients were grouped and analyzed according to their CRF subgroups, BMI was not related to outcomes.^13^

Given that the absolute risk of adverse CVD outcomes depends largely on age, employing a single relative metric cannot allow quantification of absolute risk differences across increasing ages with different BMI levels. Zaccardi et al.^14^ recently suggested a new approach to evaluate the interplay were to estimate residual life expectancy across CRF levels and BMI.^14^ Based on the large UK Biobank cohort including 474,919 participants, the authors demonstrated that participants with a brisk walking pace have a longer life expectancy across the spectrum of BMI and other fatness measures, providing additional data evidence that walking pace, as a marker of fitness level, is an easily available indicator of health status.

The strengths of the current study include the use of a clinically relevant sample based on high-risk HF patients and the assessment of CRF measures using reliable respiratory gas analysis, which is a more objective and quantitative assessment method for CRF. Several limitations include (i) the small sample size; (ii) inability to adjust for many potential confounders; (iii) lack of data on medications, biomarkers, and physical activity patterns during follow-up, which could have at least partly influenced CRF and BMI levels; (iv) lack of formal risk prediction analyses as measures of association are insufficient for making clinical judgments about the prognostic relevance of an exposure; and (v) the potential for regression dilution given the absence of repeat measurements of CRF, given the potential for changes in CRF levels due to errors in measurements and changes in the clinical course of the disease. In our reproducibility substudies of CRF measurements within the Kuopio Ischemic Heart Disease (KIHD) prospective cohort study, we have demonstrated a high within-person variability in CRF levels measured many years apart (regression dilution ratio=0.58);^15^ which suggests that analyses using only single baseline measurements of CRF could underestimate associations with outcomes. Overall, these new findings highlight the fact that an “obesity paradox” exists in HF patients and there is an interplay between obesity, CRF and outcomes in HF. Cardiorespiratory fitness as measured by VE/VCO_2_ or pVO_2_ appeared to negate the obesity paradox.^12^

CRF is influenced by genetic and environmental factors; approximately 50 % of the variation in CRF has been attributed to heritable factors, with the contribution of inherited factors to the response of CRF to physical activity and exercise training.^7^^,^^8^^,^^11^ It also depends on several factors such as baseline health and fitness status, type, duration, and intensity of PA. The level of CRF is also an indicator of a chain of multiple physiological processes that include: pulmonary ventilation and vascular function, right and left ventricular function, the capacity of the vasculature to efﬁciently transport blood from the heart to other organs matching their oxygen requirements, the ability of the muscle cells to use the oxygen and essential nutrients, and the ability to activate all necessary muscle ﬁbres needed for body movement.^11^ Left ventricular stroke volume, maximal heart rate, and arteriovenous oxygen difference at exercise have essentially determined CRF levels. Left ventricular function is a key measure of HF and CRF level may reflect LV function. As CRF is related to the integration of human body function under physiological stress conditions, it can be employed as a very accurate indicator of the risk for HF, reﬂecting whole body functional status among patients with existing HF. Given the central role normal cardiac function plays in defining maximal aerobic capacity, disease, or dysfunction that detrimentally affects cardiac output will also compromise maximal VO_2_. High-intensity aerobic exercise training is safe and effective in improving functional capacity in many patient populations with cardiac conditions.^11^ Participation in a long-term aerobic exercise training program produces a host of positive morphological and physiological cardiovascular adaptations in apparently healthy individuals, irrespective of age and sex. Commonly reported morphological adaptations associated with regular aerobic exercise training is left ventricular dilation (ie, increased end-diastolic diameter) and hypertrophy (ie, increased wall thickness), referred to as exercise training-induced cardiac remodelling.^16^

The current findings throw more light on a possible interaction between CRF, obesity and outcomes in HF. However, given the limitations of small sample size and inability to adjust for relevant covariates, these observational study findings need caution when being interpreted. Findings add to the minefield of accumulating evidence that CRF (using peak VO_2_ and measures of ventilatory efficiency) is a clinically useful tool in HF risk assessment. High levels of CRF can be achieved through regular physical activity and this should be promoted extensively via population wide approaches. The health benefits associated with regular physical activity, which includes aerobic and strength training components, cannot be overemphasized.^11^^,^^14^^,^^16^ Efforts to improve CRF with a healthy body weight could become a standard part of clinical encounters for the treatment of HF with low ejection fraction.
